# Development and internal validation of a novel predictive model for *SDHB* mutations in pheochromocytomas and retroperitoneal paragangliomas

**DOI:** 10.3389/fendo.2023.1285631

**Published:** 2023-12-21

**Authors:** Yue Zhou, Yinjie Gao, Xiaosen Ma, Tianyi Li, Yunying Cui, Yu Wang, Ming Li, Dingding Zhang, Anli Tong

**Affiliations:** ^1^ Department of Endocrinology, Key Laboratory of Endocrinology, National Health Commission of the People’s Republic of China, Peking Union Medical College Hospital, Peking Union Medical College, Chinese Academy of Medical Sciences, Beijing, China; ^2^ Department of Laboratory Medicine, Peking Union Medical College Hospital, Peking Union Medical College, Chinese Academy of Medical Sciences, Beijing, China; ^3^ Medical Research Center, State Key Laboratory of Complex Severe and Rare Diseases, Peking Union Medical College Hospital, Peking Union Medical College, Chinese Academy of Medical Sciences, Beijing, China

**Keywords:** *SDHB* mutations, pheochromocytomas and paragangliomas, prediction model, PPGLs, genetic testing

## Abstract

**Aim:**

To develop and internally validate a novel predictive model for *SDHB* mutations in pheochromocytomas and retroperitoneal paragangliomas (PPGLs).

**Methods:**

Clinical data of patients with PPGLs who presented to Peking Union Medical College Hospital from 2013 to 2022 and underwent genetic testing were retrospectively collected. Variables were screened by backward stepwise and clinical significance and were used to construct multivariable logistic models in 50 newly generated datasets after the multiple imputation. Bootstrapping was used for internal validation. A corresponding nomogram was generated based on the model. Sensitivity analyses were also performed.

**Results:**

A total of 556 patients with PPGLs were included, of which 99 had a germline *SDHB* mutation. The prediction model revealed that younger age of onset [Odds ratio (OR): 0.93, 95% CI: 0.91-0.95], synchronous metastasis (OR: 6.43, 95% CI: 2.62-15.80), multiple lesion (OR: 0.22, 95% CI: 0.09-0.54), retroperitoneal origin (OR: 5.72, 95% CI: 3.13-10.47), negative 131I-meta-iodobenzylguanidine (MIBG) (OR: 0.34, 95% CI: 0.15-0.73), positive octreotide scintigraphy (OR: 3.24, 95% CI: 1.25-8.43), elevated 24h urinary dopamine (DA) (OR: 1.72, 95% CI: 0.93-3.17), NE secretory type (OR: 2.83, 95% CI: 1.22- 6.59), normal secretory function (OR: 3.04, 95% CI: 1.04-8.85) and larger tumor size (OR: 1.09, 95% CI: 0.99-1.20) were predictors of *SDHB* mutations in PPGLs, and showed good and stable predictive performance with a mean area under the ROC curve (AUC) of 0.865 and coefficient of variation of 2.2%.

**Conclusions:**

This study provided a novel and useful tool for predicting *SDHB* mutations by integrating easily obtained clinical data. It may help clinicians select suitable genetic testing methods and make appropriate clinical decisions for these high-risk patients.

## Introduction

Pheochromocytomas and paragangliomas (PPGLs), a kind of rare neuroendocrine tumor originating from adrenal chromaffin tissue and sympathetic and parasympathetic ganglia, are recognized to have the highest heritability rate among all tumors, with almost 40% of patients found to carry germline mutations in susceptibilitygenes, including *SDHx*, *RET*, *VHL*, *FH*, *EPAS1*, and *NF1* (1–4). Among them, *SDHB* mutations are the most relevant for the treatment and prognosis of PPGLs. It is recommended that total adrenalectomy should be preferred to adrenal-sparing surgery for *SDHB*-mutated patients (5) due to their higher risk of metastasis and poorer prognosis (6–13). Therapeutic options for metastatic PPGLs are usually limited, but some retrospective clinical studies (14) have revealed that metastatic PPGLs with *SDHB* germline mutations may respond better than others to CVD (cyclophosphamide, vincristine, and dacarbazine) therapy (15–17), temozolomide (18), peptide receptor radionuclide therapy (PRRT) (19–27), and tyrosine kinase inhibitors (28–31). Importantly, patients’ *SDHB* mutations can be inherited by their offspring. It is reported that the penetrance of *SDHB*-related PPGLs is 21% by the age of 50, 42% by the age of 60 (32) and < 50% over a lifetime (33, 34). Therefore, it is necessary to identify patients with *SDHB* germline mutations as early as possible and then provide personalized management and follow-up for such high-risk patients and their families (35–37).

After the diffusion of Next Generation Sequencing (NGS), *SDHB* mutations are mainly detected through a panel of PPGL susceptibility genes, but genetic testing is expensive and time-consuming and sometimes restricted by barriers of availability, patient privacy, insurance coverage, or technical reasons. In addition, the results of genetic testing often lag behind the clinical decision, indicating that a convenient model that integrates easily obtained clinical characteristics may be useful for predicting *SDHB* mutations prior to the clinical decision itself.

Therefore, this cross-sectional study aimed to compare the clinical characteristics and prognosis between patients with and without *SDHB* mutations and to internally develop and validate a novel predictive model for *SDHB* mutations by integrating easily obtained clinical characteristics, biochemical levels, and functional imaging results.

## Methods

### Study population

Patients who presented to Peking Union Medical College Hospital from 2013 to 2022 and met the following inclusion criteria were enrolled in this study:

1. A diagnosis of pheochromocytoma or retroperitoneal paraganglioma made by experienced physicians based on clinical characteristics, biochemical tests, and functional imaging results and confirmed by pathological findings for patients undergoing surgical resection.
2. Availability of genetic tests involving the *SDHB* gene, including Sanger sequencing, a panel test of PPGL susceptibility genes, Whole Exome Sequencing (WES), and the Multiplex Ligation Dependent Probe Amplification (MLPA) test.
3. Patients with clinical information available.

Exclusion criteria were as follows:

Patients diagnosed with Multiple Endocrine Neoplasia Type 2 (MEN2) or von Hippel-Lindau (VHL) disease based on their unique clinical features and found to carry pathogenic mutations of *RET* or *VHL*, respectively.

### Data collection

Clinical data of patients were collected retrospectively without knowing their gene mutations and included sex, age of onset, age at diagnosis, presence of synchronous metastases, number and site of lesions, size and location of the primary tumor, family history of PPGLs, clinical symptoms, the highest systolic and diastolic blood pressure (SBPmax and DBPmax), information on surgery, results of immunohistochemistry (IHC) staining for SDHB and KI-67 index on resected tumors, duration of follow-up, the occurrence of recurrence and metastasis during the follow-up. Results of 24-h urinary catecholamines (CAs) tests [including norepinephrine (NE), epinephrine (E), and dopamine (DA)] and functional imaging [including 131I-meta-iodobenzylguanidine (MIBG) and octreotide scintigraphy] were also extracted.

The duration of follow-up was calculated from surgery to the last follow-up for patients undergoing surgery and from the diagnosis for those not undergoing surgery. Measurements of 24-h urinary catecholamines were done by HPLC before 01/08/2021 and by LC-MS/MS after 01/08/2021. Elevated 24-h urinary NE, E, and DA were defined as levels higher than 40.7 μg/24h, 6.4 μg/24h, and 330.5 μg/24h, respectively, when measured by HPLC and higher than 76.9 μg/24h, 11.0 μg/24h, and 459.9 μg/24h, respectively, when measured by LC-MS/MS. The secretory type was determined by the 24-h urinary CAs. If patients had elevated 24-h urinary NE but normal 24-h urinary E, their secretory type was considered NE, whereas if they had elevated 24-h urinary E regardless of NE, their secretory type was considered E (38, 39). Tumor size was calculated as the largest diameter by enhanced computed tomography (CT) or pathology results.

Metastasis was defined as the occurrence of PPGLs in distant non-chromaffin tissues, such as lung, liver, bone, and lymph nodes (40), while synchronous metastasis was defined as the occurrence of metastasis at the first diagnosis or within six months after surgical resection. Recurrence-free survival (RFS) and metastasis-free survival (MFS) were calculated from the surgery or the first diagnosis to the occurrence of recurrence and metastasis or to the last follow-up.

This study was approved by the Ethics Committee of the Peking Union Medical College Hospital in Beijing, China. Written informed consent was obtained from all the patients.

### Study design and modeling

All patients included in this study underwent germline genetic testing involving the *SDHB* gene. The genetic testing procedure has been described previously (13). The pathogenicity of *SDHB* germline mutations and deletions was classified into five grades according to the consensus of the American College of Medical Genetics and Genomics (ACMG), including “Pathogenic (P)”, “Likely Pathogenic (LP)”, “Uncertain significance (VUS)”, “Likely Benign (LB)”, and “Benign (B)”. If *SDHB* germline mutations found in patients were assessed as “P” or “LP”, their carriers could be classified into the *SDHB* group. However, if *SDHB* mutations were assessed as “VUS”, which indicated that it is unclear whether these mutations could cause PPGLs, their corresponding formalin-fixed, paraffin-embedded (FFPE) tumor tissues were used to detect the expression of SDHB, the protein encoded by the *SDHB* gene, by immunohistochemical (IHC) staining. Patients in whom the IHC on FFPE tumor tissue showed loss of SDHB protein expression were classified into the *SDHB* group, whereas patients with a preserved SDHB protein expression were classified as non-mutated. In addition, if *SDHB* mutations were assessed as “LB” or “B”, their carriers were classified into the non-*SDHB* group. Patients from both the *SDHB* group and non-*SDHB* groups were used to construct the predictive model.

MLPA testing was performed to assess the presence of *SDHB* gene deletions only in a subset of patients. If patients were found not to carry a PPGL susceptibility gene mutation but did not undergo MLPA testing and IHC staining of SDHB on FFPE tissue sections, they could not be excluded from carrying a deletion of *SDHB*, with a consequent potential misclassification bias. Therefore, to further analyze the impact of this misclassification bias on the predictive capacity of the established model, patients in the non-*SDHB* group were again divided into two groups: the identified non-*SDHB* group, with patients who underwent MLPA testing or had positive staining for SDHB on FFPE tissue sections, and the unidentified *non-*SDHB group, with patients who did not undergo MLPA testing and IHC staining for SDHB on FFPE tissue sections.

The sample size was not calculated during the study design stage but was determined by the pragmatic availability of eligible patients. Univariable logistic regression was used to recognize possible predictors of *SDHB* mutations. For missing data, the missing pattern was analyzed (**Supplementary Figure 1**; **Supplementary Table 1**). Namely, the clinical characteristics involved in the subsequent logistic regression models were compared between the groups with and without missing data. Wilcoxon and Chi-square tests, or Fisher’s exact test, were performed for continuous and categorical variables, respectively. If the results did not reach statistical significance (*P*>=0.05), their missing module was considered to be Missing Completely at Random (MCAR). Other variables (*P*<0.05) were further evaluated for the missing module. If the probability of these variables being missing could be predicted by known clinical characteristics, their missing module would be considered to be Missing At Random (MAR). After evaluation, some variables were MCAR and others were MAR. Therefore, the multiple imputation method (41–43) was used to impute these missing data, and then 50 new datasets were generated for further analysis. Subsequently, multivariable logistic models were established in 50 newly generated datasets using variables screened by backward stepwise methods (44) and clinical significance, and, finally, the coefficient of each factor was calculated as the mean of that in each model.

The model was internally validated by using the 1000-replicate bootstrapping method (45, 46) with the “mice” package. Discrimination, stability, and calibration were assessed using the mean area under the receiver operating characteristic (ROC) curve (AUC), its standard error (SD), coefficient of variation (CV), and the calibration curve. No adjustment was made to the model after evaluation, and its corresponding nomogram was built.

Moreover, the sensitivity analysis was performed to assess the predictive capacity of the model only in patients from the *SDHB* group and the identified non-*SDHB* group in order to identify the impact of patients from the unidentified non-*SDHB* group. The predictive capacity of the model was also evaluated in the dataset, where patients with missing data were excluded.

The Shapiro-Wilk test was used to assess the normality of the distribution of continuous variables. Continuous variables with a standard normal distribution were presented as mean± standard deviation (SD) and others as median (interquartile range [IQR]). Categorical variables were presented as frequencies (percentages). For all statistical analyses, a two-sided *P*<0.05 was considered statistically significant, and its 95% confidence interval (95% CI) was reported. All statistical analyses were performed using R-4.1.3 (R Foundation, www.r-project.org, Vienna, Austria).

## Results

### General characteristics of the study population

**Figure 1:**
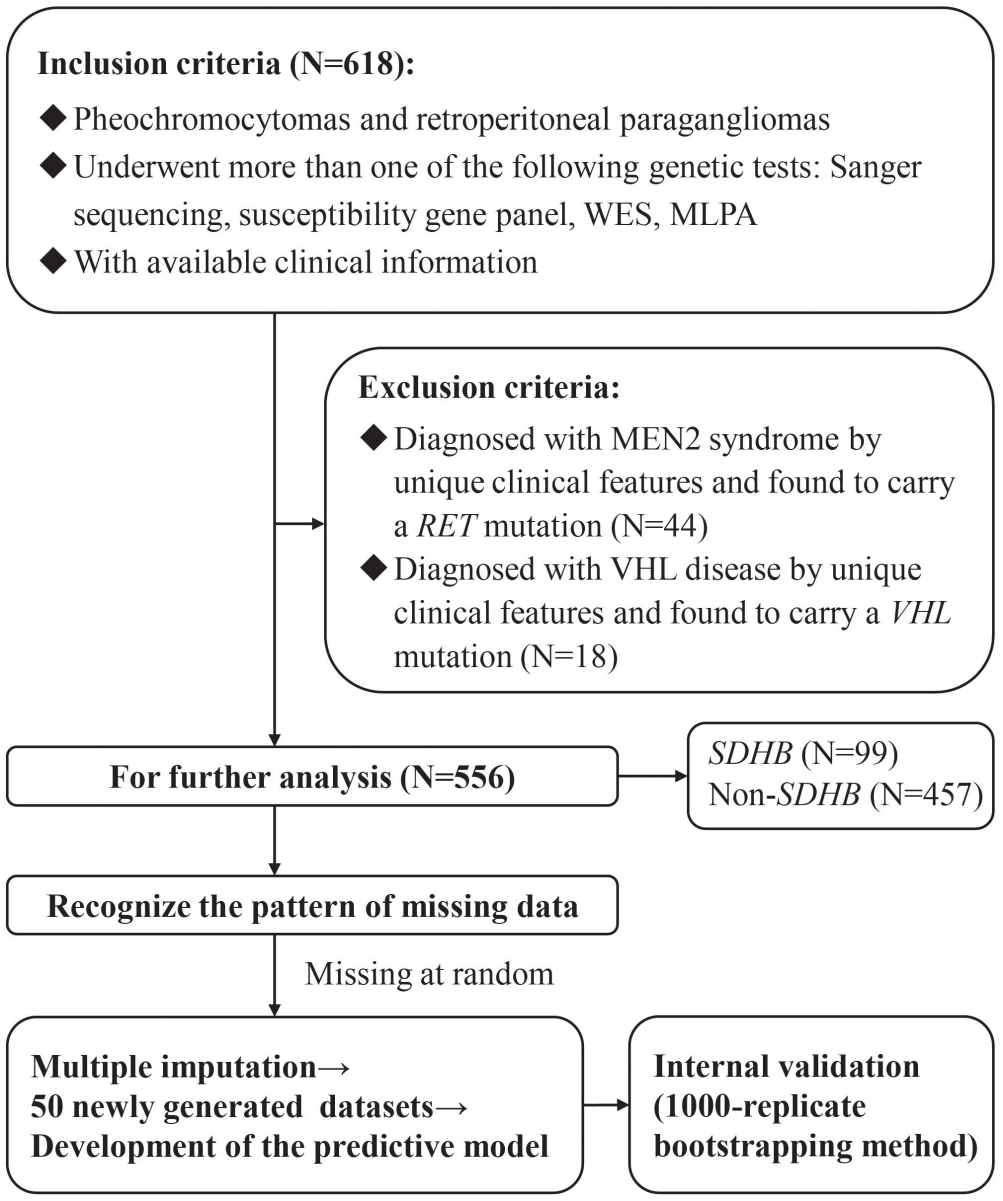
The flow diagram of the study design. WES, Whole Exome Sequencing; MLPA, Multiplex Ligation Dependent Probe Amplification; MEN2, multiple endocrine neoplasia Type 2; VHL, von Hippel-Lindau.

The study enrolled 556 patients in total (**Figure 1**), of whom 251 (45.1%) were men. The median follow-up was 4.0 (1.5-7.0) years. The median ages of onset and diagnosis were 36.5 (26.0-47.0) and 39.0 (28.0-51.0) years, respectively. A total of 419 patients (75.4%) had one of the classic symptoms, and 454 (81.7%) patients had hypertension with a median SBPmax and DBPmax of 180 (160-209) and 110 (96.75-120) mmHg, respectively. Only 13 (2.3%) patients were recorded to have a family history of PPGLs.

Tumors in 244 patients (43.9%) originated from retroperitoneal ganglia and 312 (56.1%) from adrenal glands, of which 34 (10.9%) were bilateral. The median size of the primary tumor was 5.4 (4.0-7.5) cm. In terms of functional imaging, MIBG and octreotide scintigraphy were performed in 425 (76.4%) and 433 (77.9%) patients, respectively, of which 359 (64.6%) and 328 (59.0%) showed positive results.

After the evaluation of the *SDHB* gene, 99 patients (17.8%) were classified into the *SDHB* group, and 457 (82.2%) were classified into the non-*SDHB* group, of which 290 (63.5%) were grouped into the identified non-*SDHB* group. Clinical characteristics were compared between patients in the *SDHB* and the non-*SDHB* groups, as shown in **Table 1**.

**Table 1:** The clinical characteristics of PPGLs with and without *SDHB* mutations.

	Overall (N=556)	non*SDHB* (N=457)	*SDHB* (N=99)	P-value
**Sex**				0.284
Female	305 (54.9%)	256 (56.0%)	49 (49.5%)	
Male	251 (45.1%)	201 (44.0%)	50 (50.5%)	
**Age of onset (years)**	36.5 (26.0, 47.0)	38.0 (30.0, 49.0)	25.5 (16, 37.5)	<0.001
**Age at diagnosis (years)**	39.0 (28.0, 51.0)	41.0 (31.0, 53.0)	28.0 (18.0, 42.0)	<0.001
**Familial history of PPGLs**				0.063
Yes	13 (2.3%)	8 (1.8%)	5 (5.1%)	
No	543 (97.7%)	449 (98.2%)	94 (94.9%)	
**Classic symptoms**				0.068
Yes	419 (75.4%)	352 (77.0%)	67 (67.7%)	
No	137 (24.6%)	105 (23.0%)	32 (32.3%)	
**Hypertension**				0.822
Yes	454 (83.9%)	373 (84.2%)	81 (82.7%)	
No	87 (16.1%)	70 (15.8%)	17 (17.3%)	
**SBPmax (mmHg)**	180 (160, 209)	180 (160, 210)	180 (160, 200)	0.212
**DBPmax (mmHg)**	110 (96.75, 120)	110 (92, 120)	120 (100, 130)	0.019
**Origin site**				<0.001
Adrenal PHEOs	312 (56.1%)	285 (62.4%)	27 (27.3%)	
Retroperitoneal PGLs	244 (43.9%)	172 (37.6%)	72 (72.7%)	
**Bilateral/Unilateral adrenal**				<0.001
Bilateral	34 (10.9%)	34 (11.9%)	0 (0%)	
Unilateral	278 (89.1%)	251 (88.1%)	27 (100.0%)	
**Multiple lesions**				0.094
Yes	77 (13.8%)	69 (15.1%)	8 (8.1%)	
No	479 (86.2%)	388 (84.9%)	91 (91.9%)	
**Primary tumor size (cm)**	5.4 (4.0, 7.5)	5.2 (4.0, 7.0)	6.0 (5.0, 8.0)	0.002
**Surgery**				0.627
Yes	519 (93.5%)	428 (93.9%)	91 (91.9%)	
No	36 (6.5%)	28 (6.1%)	8 (8.1%)	
**Duration of follow-up (years)**	4.0 (1.5, 7.0)	4.0 (1.5, 7.0)	4.0 (1.8, 6.0)	0.915
**Synchronous metastasis**				<0.001
Yes	37 (6.7%)	16 (3.5%)	21 (21.2%)	
No	518 (93.3%)	440 (96.5%)	78 (78.8%)	
**Metastasis**				<0.001
Yes	176 (31.7%)	108 (23.7%)	68 (68.7%)	
No	379 (68.3%)	348 (76.3%)	31 (31.3%)	
**Recurrence**				0.049
Yes	134 (24.1%)	102 (22.4%)	32 (32.3%)	
No	421 (75.9%)	354 (77.6%)	67 (67.7%)	
**MIBG**				<0.001
Positive	359 (84.5%)	299 (87.7%)	60 (71.4%)	
Negative	66 (15.5%)	42 (12.3%)	24 (28.6%)	
**Octreotide scintigraphy**				<0.001
Positive	328 (75.8%)	253 (72.1%)	75 (91.5%)	
Negative	105 (24.2%)	98 (27.9%)	7 (8.5%)	
**Elevated DA**				0.068
Elevated	137 (27.2%)	105 (25.4%)	32 (35.6%)	
Normal	366 (72.8%)	308 (74.6%)	58 (64.4%)	
**Secretory type**				<0.001
E	136 (25.9%)	127 (29.5%)	9 (9.4%)	
NE	310 (58.9%)	240 (55.8%)	70 (72.9%)	
Normal	80 (15.2%)	63 (14.7%)	17 (17.7%)	
**Ki-67 index on IHC (%)**	2.0 (1.0, 5.0)	2.0 (1.0, 3.0)	3.0 (2.0, 8.0)	<0.001

PPGLs, pheochromocytomas and paragangliomas; SBPmax, the maximum of systolic blood pressure; DBPmax, the maximum of diastolic blood pressure; MIBG, 131I-meta-iodobenzylguanidine; DA, dopamine; E, epinephrine; NE, norepinephrine; IHC, immunohistochemistry.

All continuous variables were non-normally distributed and presented as median [interquartile range (IQR)], and categorical variables as frequency (percentage). The Wilcoxon tests were used to compare the differences in continuous variables between patients with and without a germline SDHB-mutation, while the Chi-Squared tests or Fisher’s exact tests were used for categorical variables.

If patients had elevated 24h urinary NE but normal 24h urinary E, their secretory type was considered as NE, whereas if they had elevated 24h urinary E regardless of NE, their secretory type was considered as E.

### Prognosis of patients with and without *SDHB* mutations

**Figure 2:**
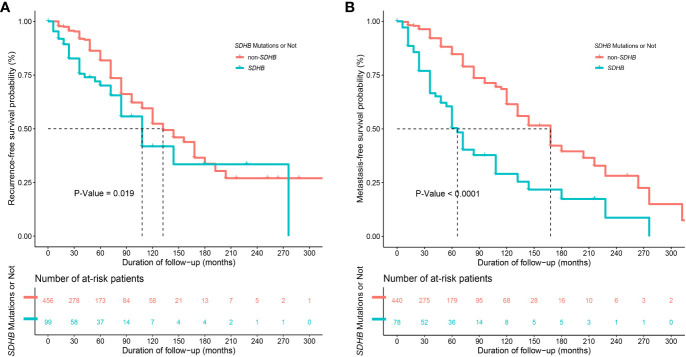
Kaplan-Meier curves of **(A)** recurrence-free survival (RFS)" between patients with and without *SDHB* germline mutations (with only 555 patients' information available) and of **(B)** metastasis-free survival (MFS) between patients with and without *SDHB* germline mutations (with only 518 patients' information available after excluding 37 patients with synchronous metastases).

Synchronous metastases were found in 37 (6.7%) patients. During the follow-up, 134 (24.1%) and 139 (25.0%) patients developed recurrence and distant metastasis, respectively. Compared with patients in the non-*SDHB* group, those in the *SDHB* group had worse RFS (*P*=0.019, **Figure 2A**) and MFS (*P*<0.001, **Figure 2B**), as well as a higher KI67 index [3.0% (2.0-8.0) vs. 2.0% (1.0- 3.0), *P*<0.001], which was regarded as a risk factor for poor prognosis.

### Development and internal validation of the predictive model for *SDHB* mutations

Univariable logistic analysis revealed that younger age of onset [odds ratio (OR): 0.94, 95% CI: 0.93-0.96; *P*<0.001], synchronous metastasis (OR: 7.4, 95% CI: 3.72-15.02; *P*<0.001), retroperitoneal origin (OR: 4.42, 95% CI: 2.76-7.25; *P*<0.001), negative MIBG (OR: 0.35, 95% CI: 0.2-0.63; *P*<0.001), positive octreotide scintigraphy (OR: 4.15, 95% CI: 1.97-10.19; *P*<0.001), secretory type of NE [OR: 4.12 (compared with secretory type of E), 95% CI: 2.09-9.09; *P*<0.001], normal 24-h urinary E and NE [OR: 3.81 (compared with secretory type of E), 95% CI: 1.64-9.39; *P*=0.002], larger primary tumor size (OR: 1.09, 95% CI: 1.02-1.17; *P*=0.009), absence of classic symptoms (OR: 0.62, 95% CI: 0.39-1.01; *P*=0.052), elevated urinary DA (OR: 1.62, 95% CI: 0.99-2.62; *P*=0.052), family history of PPGLs (OR: 2.99, 95% CI: 0.89-9.15; *P*=0.060), higher DBPmax (OR: 1.01, 95% CI: 1-1.02; *P*=0.061), and single lesion (OR: 0.49, 95% CI: 0.21-1.01; *P*=0.072) were predictors of *SDHB* mutations (**Table 2**).

**Table 2:** Univariate and multivariate logistic regression analysis of predictors for PPGLs with *SDHB* mutations.

	Univariable logistic regression analysis				Multivariable logistic regression analysis		
Variables	Sample size	OR	95% CI	*P*-Value	OR	95% CI	*P*-Value
**Male**	556	1.30	0.84-2.01	0.238	–	–	–
**Age of onset**	556	0.94	0.93-0.96	<0.001	0.93	0.91-0.95	<0.001
**With familial history of PPGLs**	556	2.99	0.89-9.15	0.060	–	–	–
**With classic symptoms**	556	0.62	0.39-1.01	0.052	–	–	–
**With hypertension**	541	0.89	0.51-1.64	0.706	–	–	–
**SBPmax**	520	1.00	0.99-1.00	0.167	–	–	–
**DBPmax**	500	1.01	1.00-1.02	0.061	–	–	–
**With multiple lesions**	556	0.49	0.21-1.01	0.072	0.22	0.09-0.54	0.001
**With synchronous metastasis**	555	7.40	3.72-15.02	<0.001	6.43	2.62-15.8	<0.001
**Retroperitoneal origin site**	556	4.42	2.76-7.25	<0.001	5.72	3.13-10.47	<0.001
**Positive MIBG**	425	0.35	0.20-0.63	<0.001	0.34	0.15-0.73	0.006
**Positive octreotide scintigraphy**	433	4.15	1.97-10.19	0.001	3.24	1.25-8.43	0.016
**Elevated DA**	503	1.62	0.99-2.62	0.052	1.72	0.93-3.17	0.083
**Secretory type of normal E and NE**	526	3.81	1.64-9.39	0.002	3.04	1.04-8.85	0.041
**Secretory type of NE**	526	4.12	2.09-9.09	<0.001	2.83	1.22-6.59	0.016
**Primary tumor size**	500	1.09	1.02-1.17	0.009	1.09	0.99-1.20	0.075

OR, odds ratio; 95% CI, 95% confidence interval; SBPmax, the maximum of systolic blood pressure; DBPmax, the maximum of diastolic blood pressure; MIBG, 131I-meta-iodobenzylguanidine; DA, dopamine; E, epinephrine; NE, norepinephrine; MIBG, 131I-meta-iodobenzylguanidine. Multivariate logistic regression analysis was performed in 50 newly generated datasets using variables screened by the backward stepwise methods as well as clinical significance. Finally, the coefficient of each factor was calculated as the mean of that in each model. The OR of the secretory type of NE and the secretory type of normal E and NE was calculated relative to the secretory of E.

In the multivariable logistic model, younger age of onset (OR: 0.93, 95% CI: 0.91-0.95; *P*<0.001), synchronous metastasis (OR: 6.43, 95% CI: 2.62- 15.80; *P*<0.001), single lesion (OR: 0.22, 95% CI: 0.09-0.54; *P*<0.001), retroperitoneal origin (OR: 5.72, 95% CI: 3.13-10.47; *P*<0.001), negative MIBG (OR: 0.34, 95% CI: 0.15-0.73; *P*=0.006), positive octreotide scintigraphy (OR: 3.24, 95% CI: 1.25-8.43; *P*=0.016), elevated 24-h urinary DA (OR: 1.72, 95% CI: 0.93-3.17; *P*=0.083), secretory type of NE [OR: 2.83 (compared with the secretory type of E), 95% CI: 1.22- 6.59; *P*=0.016], normal 24h urinary E and NE [OR: 3.04 (compared with the secretory type of E), 95% CI: 1.04-8.85; *P*=0.041], and larger primary tumor size (OR: 1.09, 95% CI: 0.99-1.20; *P*=0.075) were independent predictors of *SDHB* mutations (**Table 2**).

**Figure 3:**
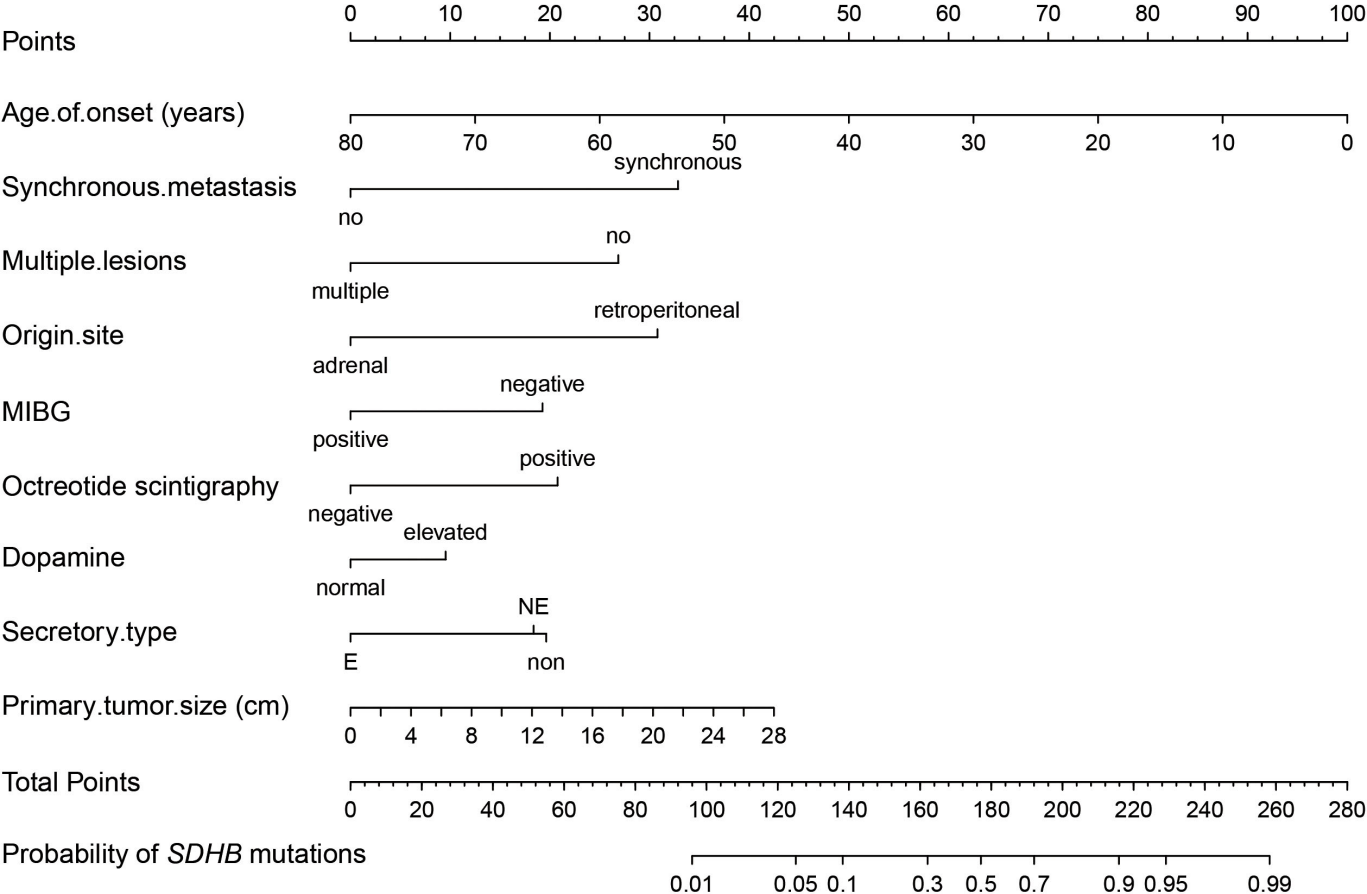
Nomogram of the established prediction model for *SDHB* mutations. MIBG, 131I-meta-iodobenzylguanidine; If patients had elevated 24-h urinary NE but normal 24-h urinary E, their secretory type was considered to be NE, whereas if they had elevated 24-h urinary E regardless of NE, their secretory type was considered to be E.

The model showed rather good discrimination with an AUC of 0.866 (95% CI: 0.828-0.903) (**Supplementary Figure 2A**) and calibration (R^2^ = 0.417, *P* = 0.775) (**Supplementary Figure 2B**) in a randomly selected dataset. After internal validation with 1000 bootstrap samples, the model still showed good discrimination with a mean AUC of 0.865 and good stability with an SD and CV of the AUC of 0.019 and 2.2%, respectively. Therefore, the model was not adjusted and was presented as a nomogram (**Figure 3**).

The results of the sensitivity analysis revealed an AUC of 0.873 (95% CI: 0.834-0.912) in 389 patients after excluding 167 unidentified non-*SDHB* patients and 0.871 (95% CI: 0.822-0.921) in 324 patients after excluding those with missing data.

## Discussion

This cross-sectional study retrospectively summarized the differences in clinical characteristics and prognosis between patients with and without *SDHB* mutations based on a large population of 556 PPGL cases. Importantly, we proposed a novel prediction model for *SDHB* mutations that integrates easily obtained clinical characteristics, functional imaging results, and biochemical parameters and showed good predictive performance and stability. The corresponding nomogram will be convenient and informative for clinicians to make an appropriate clinical decision for these high-risk patients when genetic testing is not available or the report is delayed.

Our results are consistent with the previous findings revealing that *SDHB*-mutated PPGLs are more likely to originate from extra-adrenal locations (13, 47, 48), have a younger age of onset (48), have the secretory type of NE (8), have a larger size (8), and express high levels of SSTR2 (19, 49). In addition, previous studies have mostly focused on the differences between pseudohypoxia type (PHT) PPGLs and non-PHT PPGLs and found that most PHT PPGLs presented with a younger age of onset (50), a noradrenergic phenotype (51, 52), and elevated DA (52). A recent study also developed a nomogram based on age ≤35 years, hypertension, 24-h urinary vanillylmandelic acid output (VMA) ≥100 umol/24-h and urinary 17-ketosteroid (17 KS) ≤50 umol/24-h levels to discriminate PHT from non-PHT PPGLs, which showed good discriminatory performance with an AUC of 0.829 (95% CI, 0.767–0.891) (53). However, hypertension was not regarded as a significant predictor in our study, possibly because it is a relatively nonspecific clinical finding (14). Another study used pre-operative weight loss > 10% body weight, elevated pre-operative hematocrit > 50%, normal baseline heart rate < 100 bpm, and normal plasma metanephrines < 0.60 nmol/L to predict the PHT PCCs and achieved an AUC of 0.831 (50). However, the above parameters were not collected in the study design, so we could not assess their predictive performance in our patients.

Our study also demonstrated that PPGLs with *SDHB* mutations had a higher proportion of recurrence and metastasis and a poorer MFS and RFS, as commonly reported in other studies (48). It is suggested that, compared with other genotypes, PPGLs with *SDHB* mutations should have more frequent and longer follow-ups and a more aggressive surveillance plan due to their poorer prognosis.

However, there were also some limitations in our study. First, as some patients were not initially diagnosed at our hospital, the clinical characteristics of their primary tumors were partially missing. However, after excluding patients with missing data, the results of the sensitivity analysis showed good predictive performance with an AUC of 0.871 (95% CI: 0.822- 0.921), indicating the low impact of missing data on the discrimination of the model. Second, among the patients in the non-*SDHB* group, 167 were grouped into the unidentified non-*SDHB* group due to the uncertainty of *SDHB* gene deletion, which might cause misclassification bias. However, the model also showed a good performance with an AUC of 0.873 (95% CI: 0.834- 0.912) in predicting *SDHB* mutations when considering only the 389 patients from the *SDHB* group and the identified non-*SDHB* group to exclude the influence of a potential misclassification bias. In fact, deletion in *SDHB* is rare in the Chinese population, with only 1.6% (1/61) described in our previous study (13). Third, our study only included patients with retroperitoneal paragangliomas and pheochromocytomas, but not those with paragangliomas originating from other organs, such as the head and neck [mostly associated with *SDHD* mutations and less commonly with *SDHB* mutations (54)], mediastinum, pelvic cavity, and bladder. For this reason, the application of the predictive model proposed in this study may be limited to retroperitoneal pheochromocytomas and PGLs only, which, however, represent the vast majority of PPGLs. In addition, the model proposed in this study included 24-h urinary CAs and octreotide scintigraphy, which have been progressively replaced in clinical practice by plasma-free metanephrines and 24-h urinary fractionated metanephrines, and PET/CT with 68Ga-somatostatin analogs (68Ga-SSA) due to their higher sensitivity and accuracy in the diagnosis of PPGLs. However, these data were only available in a few patients, which prevented their application in the predictive model construction. It is possible that the use of these parameters will further improve the discrimination of the model. Finally, this is a retrospective and single-center study that only included patients who underwent genetic testing, so selection bias may exist. Although our model included a large population and showed relatively good predictive performance, external validation is still required.

## Conclusions

This retrospective study proposed a convenient and informative tool for predicting *SDHB* mutations in pheochromocytoma and retroperitoneal PGLs by integrating easily obtained clinical, biochemical, and functional imaging characteristics. The proposed model could help clinicians make an appropriate decision and plan a personalized follow-up for these high-risk patients.

## Data availability statement

The dataset included privacy clinical information of patients. Requests to access these datasets should be directed to tongal@pumch.cn.

## Ethics statement

This study was approved by the Ethics Committee of the Peking Union Medical College Hospital in Beijing, China. Written informed consent was obtained from all the patients.

## Author contributions

YZ: Data curation, Formal analysis, Software, Validation, Visualization, Writing – original draft, Writing – review & editing. YG: Data curation, Project administration, Resources, Writing – review & editing. XM: Data curation, Resources, Writing – review & editing. TL: Data curation, Resources, Writing – review & editing. YC: Data curation, Investigation, Project administration, Writing – review & editing. YW: Investigation, Resources, Writing – review & editing. ML: Methodology, Resources, Writing – review & editing. DZ: Conceptualization, Methodology, Software, Supervision, Validation, Writing – review & editing. AT: Conceptualization, Funding acquisition, Project administration, Resources, Supervision, Writing – review & editing.
