# Supplementary material for: Development and internal validation of a novel predictive model for SDHB mutations in pheochromocytomas and retroperitoneal paragangliomas
Source: Front Endocrinol (Lausanne). 2023 Dec 21;14:1285631. doi: 10.3389/fendo.2023.1285631 (PMC10764617; doi:10.3389/fendo.2023.1285631)
Supplement: Supplementary file 1 [file Image_1.pdf]

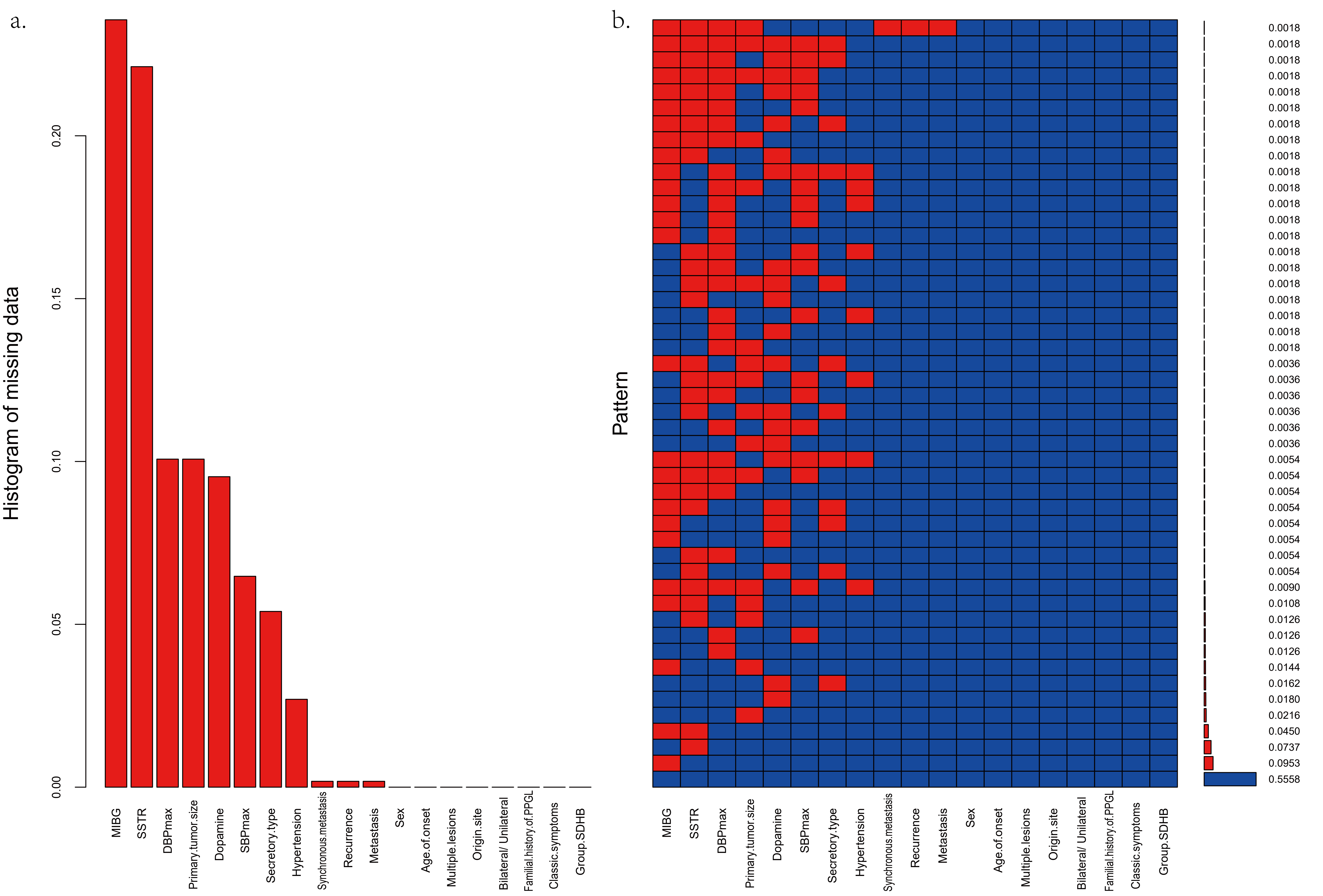

The Supplemental Figure 1. a. The percentage of missing data of each variable; **b.** The missing pattern of each variable and the percentage of patients with different missing patterns.
