# Supplementary material for: Development and internal validation of a novel predictive model for SDHB mutations in pheochromocytomas and retroperitoneal paragangliomas
Source: Front Endocrinol (Lausanne). 2023 Dec 21;14:1285631. doi: 10.3389/fendo.2023.1285631 (PMC10764617; doi:10.3389/fendo.2023.1285631)
Supplement: Supplementary file 2 [file Image_2.pdf]

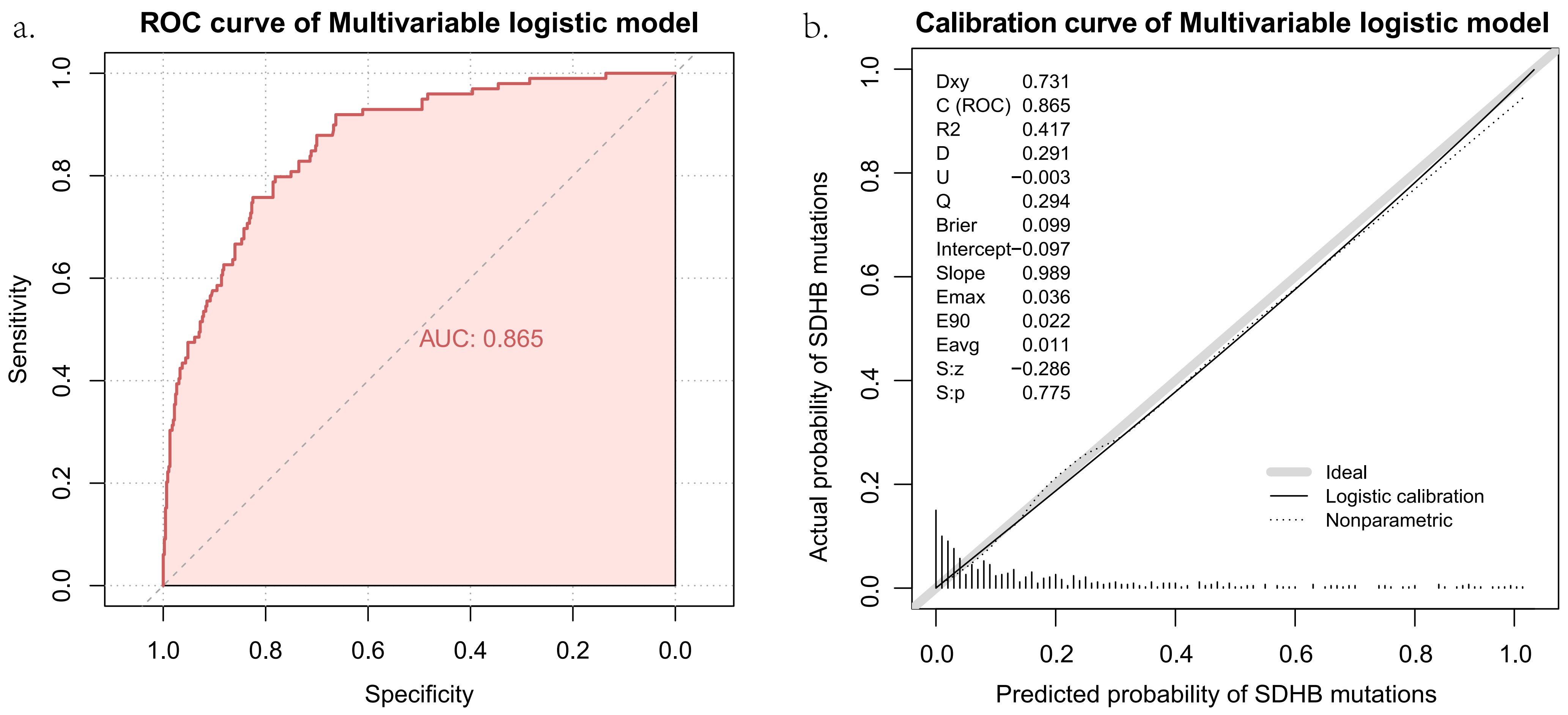

The Supplemental Figure 2. a. The ROC curve and its AUC and **b.** the calibration curve of the established multivariable logistic model evaluated in a randomly selected dataset after multiple imputation.
